# Toward One Health: a spatial indicator system to model the facilitation of the spread of zoonotic diseases

**DOI:** 10.3389/fpubh.2023.1215574

**Published:** 2023-06-29

**Authors:** Daniel Jato-Espino, Fernando Mayor-Vitoria, Vanessa Moscardó, Fabio Capra-Ribeiro, Leticia E. Bartolomé del Pino

**Affiliations:** ^1^GREENIUS Research Group, Universidad Internacional de Valencia—VIU, Calle Pintor Sorolla, Valencia, Spain; ^2^School of Architecture, College of Art and Design, Louisiana State University, Baton Rouge, LA, United States

**Keywords:** geographic information system, multi-criteria decision-making, green infrastructure, indicators, one health, systematic literature review

## Abstract

Recurrent outbreaks of zoonotic infectious diseases highlight the importance of considering the interconnections between human, animal, and environmental health in disease prevention and control. This has given rise to the concept of One Health, which recognizes the interconnectedness of between human and animal health within their ecosystems. As a contribution to the One Health approach, this study aims to develop an indicator system to model the facilitation of the spread of zoonotic diseases. Initially, a literature review was conducted using the Preferred Reporting Items for Systematic Reviews and Meta-Analyses (PRISMA) statement to identify relevant indicators related to One Health. The selected indicators focused on demographics, socioeconomic aspects, interactions between animal and human populations and water bodies, as well as environmental conditions related to air quality and climate. These indicators were characterized using values obtained from the literature or calculated through distance analysis, geoprocessing tasks, and other methods. Subsequently, Multi-Criteria Decision-Making (MCDM) techniques, specifically the Entropy and Technique for Order of Preference by Similarity to Ideal Solution (TOPSIS) methods, were utilized to combine the indicators and create a composite metric for assessing the spread of zoonotic diseases. The final indicators selected were then tested against recorded zoonoses in the Valencian Community (Spain) for 2021, and a strong positive correlation was identified. Therefore, the proposed indicator system can be valuable in guiding the development of planning strategies that align with the One Health principles. Based on the results achieved, such strategies may prioritize the preservation of natural landscape features to mitigate habitat encroachment, protect land and water resources, and attenuate extreme atmospheric conditions.

## 1. Introduction

Zoonotic diseases pose a significant public health concern, with over 70% of emerging diseases being transmitted from animals to humans and 60% of human infectious diseases being shared with animals (1). This means that zoonotic diseases have played a role in recent outbreaks, including Ebola and coronavirus pandemics, as well as in well-known foodborne illnesses. These diseases can be transmitted not only through direct contact with animals or vectors, or the ingestion of animal products, but also through the consumption of contaminated vegetables grown in areas where domestic or wild animal manure or irrigation water is used (2).

The transmission of zoonotic diseases is a complex and multifactorial process, making it difficult to manage and predict due to the interconnected elements involved. Therefore, an interdisciplinary approach is necessary; focusing not only on disease surveillance but also on the development of predictive models (3).

The concept of One Health is crucial for today’s sustainable human development. One Health is “an approach that recognizes people’s health, closely connected to the health of animals and our shared environment” (4). The recurrence of outbreaks of emerging and re-emerging zoonotic diseases has emphasized the importance of the One Health approach, which acknowledges the interconnectedness of human, animal, and environmental health (5, 6). Achieving One Health requires a comprehensive understanding of the complex interactions and feedback between these systems and the identification of interventions that can promote positive outcomes for all (7). However, measuring progress toward One Health goals remains challenging, and various initiatives have employed different indicator systems to address this issue (8–10).

There is an ongoing debate on the most effective way to measure progress toward One Health goals (11–14), but there is a clear relationship between contagious diseases and spatial configurations and conditions (15, 16). The exponential growth of human population has disrupted the interface between humans, animals, and the environment through increased urbanization and the expansion of livestock and agricultural areas (17). These processes lead to the fragmentation of wildlife habitats that increase interspecies friction and the spread of pathogens. For example, it has been argued that certain infectious diseases are exacerbated by factors such as rapid urbanization, large migrant workers populations, climate change, ecological changes, and policies like deforestation (18).

Spatial assessment of disease susceptibility or transmissibility is crucial for the One Health approach as it helps identify areas where zoonotic diseases are more likely to occur and spread. Understanding the relationship between different spatial configurations and the spread of pathogens is essential to reduce the transmission of infectious diseases (17, 19, 20). However, it is a complex and challenging subject.

The number of publications examining the relationship between spatial configuration and the spread of infectious diseases increased from 100 in 2000 to over 700 publications in 2017 (20). This highlights the growing interest in this field and the motivation behind conducting a systematic literature review to better understand what had been done previously.

Previous research has addressed this issue from different perspectives. There is a general trend toward interdisciplinary strategies (17), although the focus has often been on program implementation rather than contextual research (21). More specifically, the analysis diverges in multiple directions. The interest in modeling methodologies has been of particular interest, but different approaches have been taken. Some authors have incorporated census data, land use information, and population mobility into their model design (22), while others have examined multiple cases to understand how mathematical models can generate robust evidence and shape effective public health policies at local and global levels (23). Recent research shows a notable trend toward automation and the development of more complex models.

Significant research has attempted to understand how proximity to Green Infrastructure (GI), which refers to a network of natural and semi-natural areas, can help improve human health (24). Other studies have focused on zoonoses related to ecosystems, assessing their impact on human health while examining the existing evidence of ecological responses to global changes (25). Recent studies have emphasized the need for a holistic approach within the concept of One Health to predict and prevent future pandemics (26, 27). Some models have considered different spatial conditions (28), while others have focused on specific species (29). An important percentage analyzed specific outbreaks in detail (e.g. (30–33),). In these cases, the proliferation of Geographic Information Systems (GIS) has contributed to a better understanding of the role of space in pathogen spillover (34, 35). For more details, refer to (36–39).

In general, the study of previous research has provided insights into the strong relationship between the spread of diseases and various spatial conditions. However, we could not find any studies specifically addressing how these spatial conditions could represent susceptibility or weaknesses that contribute to the faster, stronger, or broader spread of zoonotic diseases. Apart from the previously mentioned approaches, some authors have worked on developing indicators to better understand One Health conditions (8, 9, 40), but they used qualitative research based on binary logic, were conducted at the regional/national scale, or were somehow not holistic and complex enough to capture all the conditions prevailing in the area. In other words, to address a given area using the One Health framework, holistic tools to assess its spatial susceptibility need to be developed.

This research helps to fill this knowledge gap by developing a spatial indicator system that models the facilitation of zoonotic diseases spread, providing insights into a region’s contribution to One Health. The proposed indicator system takes a multidimensional approach for One Health, incorporating indicators of human, animal, and environmental health, as well as their interactions. We apply this indicator system to the region of Valencia, Spain, which exhibits diverse ecosystems and land uses including agriculture, urban areas, and natural areas. The novelty of this research lies in the development of a practical tool for measuring a region’s contribution to One Health.

As mentioned earlier, One Health requires collaboration among different sectors and stakeholders (41–43). This case study aims to provide policymakers, researchers, and practitioners with a practical tool for monitoring progress toward One Health goals and identifying areas for intervention and improvement. Furthermore, our study seeks to evaluate the usefulness and validity of the proposed indicator system by comparing its results with infectious disease records in the case study area and assessing the potential role of GI in achieving One Health.

This document is structured as follows: first, we review the literature on One Health and indicator systems. Second, we describe the methodology employed to develop the indicator system and apply it to the region of Valencia, Spain. Third, we present the results of our case study and evaluate the utility and validity of the proposed indicator system. Finally, we discuss the implications of our findings and provide recommendations for future research.

## 2. Materials and methods

**Figure 1:**
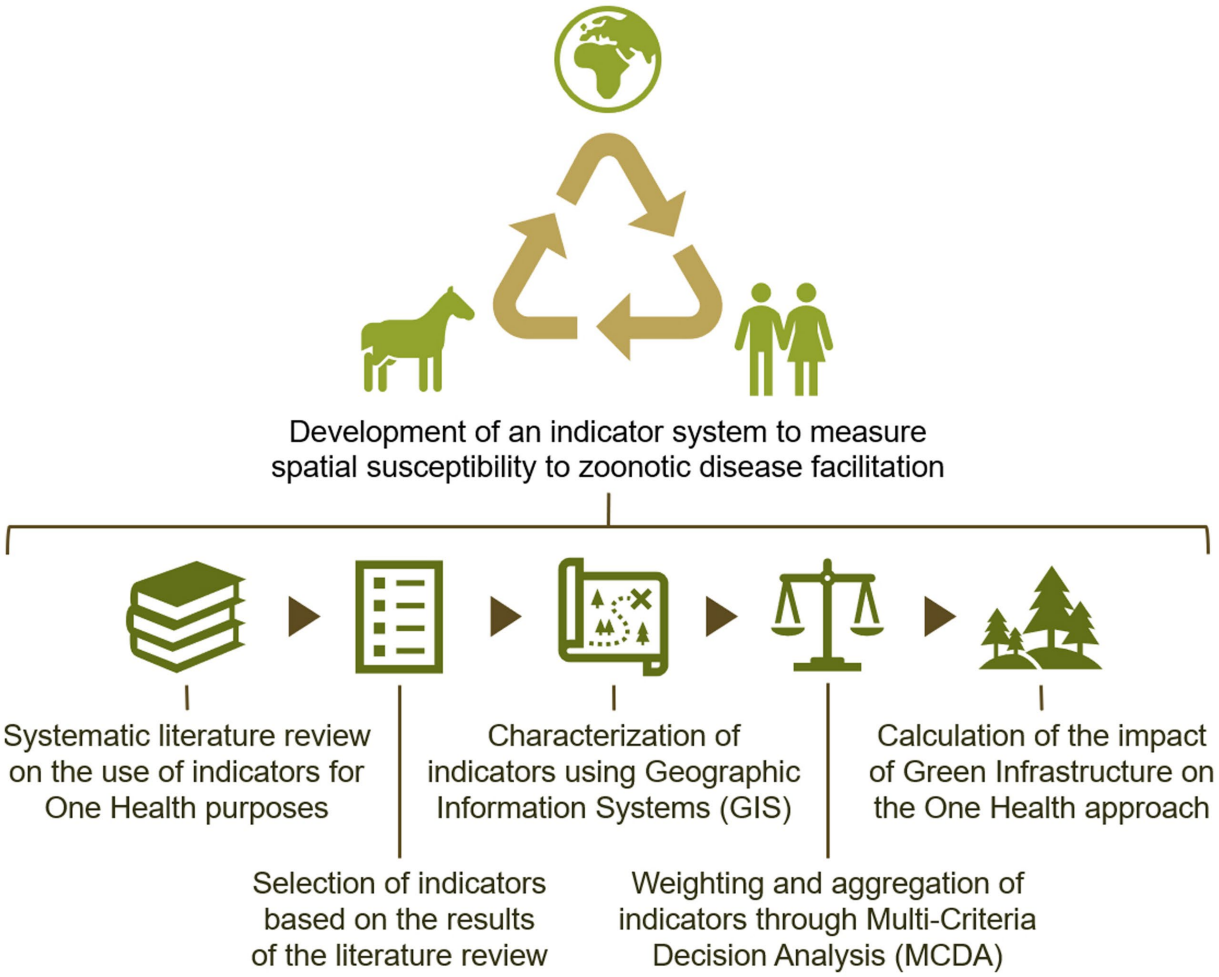
Components of the indicator system to measure the facilitation of the spread of zoonotic diseases.

The steps for designing and applying the proposed indicator system are depicted in Figure 1. A systematic literature review was first conducted to gain insights into the breadth of prior research on using indicators for One Health goals. The outcomes of the literature review were used to select a list of indicators that encompassed aspects related to animal, human, and environmental health. GIS and Multi-Criteria Decision-Making (MCDM) methods were employed to characterize, weight, and aggregate the shortlisted indicators. This resulted in a composite index that reflects the contributions of a region’s spatial context to One Health. These index values were then compared with the area covered by GI to assess its impact on One Health.

### 2.1. Selection of indicators

The indicators were selected based on the results of a literature review conducted according to the Preferred Reporting Items for Systematic Reviews and Meta-Analyses (PRISMA) statement (44). The literature review aimed to answer the research question: are there any indicators or metrics that have been consistently used to address One Health issues? Finding an answer to this question should result in a set of indicators ranked by their frequency of use in previous research. The inclusion criteria for the review were as follows:

- The publications are original research articles.
- The articles are indexed in the Scopus, Web of Science or PubMed databases.
- The year of publication of the documents is equal to or later than 2004.
- The articles are published in English.

Review articles, conference papers, and books were excluded from the eligible items to focus on original research contributions that utilized indicators to address the One Health initiative. The search included the Scopus, Web of Science, and PubMed databases because of their extensive journal coverage, temporal range, and relevance to medical research, respectively (45). The time frame was limited to 2004 onwards, aligning with the year when the term One Health was coined (46, 47). The documents had to be written in English because of its consideration as the language of science (48).

The search query included the terms “one health,” and either “indicator*” or “metric*” in the title, abstract, or keywords of the documents. Additional specific terms related to different facets of the One Health concept, particularly those with spatial implications, were also incorporated into the search query. Eq. (1) presents the search query used in the Scopus database.

TITLE-ABS-KEY [“one health” AND (“indicator*” OR “metric*”)] AND [“agricultur*” OR “air” OR “biodiversity” OR “climate change*” OR “disease*” OR “ecologic*” OR “ecosystem*” OR “epidemi*” OR “food” OR “forest*” OR “habitat” OR “land use*” OR “livestock” OR “pollution” OR “population” OR “soil” OR “urban*” OR “warming” OR “water” OR “wildlife”] AND LIMIT-TO (DOCTYPE, “ar”)] AND [LIMIT-TO (PUBYEAR, 2022–2004) AND LIMIT-TO (LANGUAGE, “English”]. (1)

The results from the databases were combined to eliminate duplicates. Aside from the fields returned directly from the query (authors, year, title, abstract, and keywords), a few others were added manually to gather more specific information. One of these fields was used to track the reasons for discarding initially eligible articles. This could be for a variety of reasons, including the article being a review that is not labeled as such, misinterpretation of search query terms (e.g., “one’s health” instead of “one health”), or the absence of suggested indicators. Invalid documents could be identified through reading the abstracts or full texts.

Six additional fields were related to the dimensions of One Health. Three fields indicated whether articles emphasized animal, human, or environmental health, while the other three fields recorded the specific terms used for each dimension. Two binary fields were included to indicate whether the articles considered GI and whether they had a spatial component. Another pair of fields compiled the indicators proposed in the papers and derived from reading them.

The screened documents were processed to generate frequency counts from the words contained in some fields, particularly keywords, and indicators, as well as the binary data on the dimensions of One Health and the presence of GI and spatial approaches. After reclassification and standardization, this analysis revealed the main trends found in the articles and created a hierarchy of the most frequently recurring indicators for modeling One Health.

### 2.2. Processing of indicators

Due to the orientation of the study on the role of spatial planning in the facilitation of zoonotic disease spread, the indicators were designed in a way that allowed them to be characterized using GIS. This involved applying geoprocessing tools to conduct spatial analyses regarding densities, distances, or algebraic operations. The resulting maps were then used to calculate descriptive statistics (mean or sum) per administrative unit through zonal calculations.

The outputs of this characterization process enabled the creation of a matrix of m indicators assessed with $x_{ij}$ values across n administrative units. This arrangement resembles an MCDM problem, where multiple alternatives are evaluated depending on a set of criteria (49). MCDM problems broadly consist of two steps: weighting of criteria (indicators) and assessment of alternatives (administrative units) across the weighted criteria (50). Given the multiple branches that stem from the concept of One Health, the selection of indicators aimed to capture different aspects of environmental, human, and animal health.

#### 2.2.1. Weighting of indicators

The weighting of indicators was performed using the Entropy Method (EM), which was proposed by Zeleny (51) to objectively calculate the weights of criteria in decision-making processes. The importance of a criterion is assumed to be proportional to the amount of information it provides about the alternatives. The idea is to give more weight to the criterion that can better discriminate the other options, i.e., the criterion that shows greater diversity when evaluating the other options. The higher the entropy ( $E_{j}$ _j_), the lower the diversity ( $1-E_{j}$ ).

In this study, the EM was employed to determine the weights of indicators based on their differentiation. Indicators that exhibited more distinct values across the administrative units contained more information and had lower entropy (52). This implies large weights for indicators with low entropy values and vice versa. For example, if all administrative units had very similar values (e.g., from 0.5 to 0.6) regarding a particular indicator, that indicator would be given a low weight. Conversely, if another indicator exhibited a wide range of values (e.g., from 0.1 to 0.9) across the administrative units, it would be assigned a higher weight.

To enable a proper comparison of the index dimensions within the decision-making matrix a max-min transformation was applied to normalize the values $x_{ij}$ of the m indicators across the n administrative units, as shown in Eq. (2). Transformation ensured that the indicator values were on a comparable scale.


(2) $$r_{ij}=\{\begin{array}{c} \frac{x_{ij}-\min x_{j}\;}{\max x_{j}-\min x_{j}\;},forbenefitindicators\\ \frac{\max x_{j}-x_{ij}}{\;\max x_{j}-\min x_{j}},forcostindicators \end{array}$$

where $\max x_{j}$ and $\min x_{j}$ are the maximum and minimum values among the alternatives for indicator j . The entropy $E_{j}$ of each indicator was determined from the normalized values $r_{ij}$ as formulated in Eq. (3).


(3) $$E_{j}=-\frac{\sum_{i=1}^{n}f_{ij}\;\cdot \;\ln f_{ij}}{\ln n}\;\left(i=1,\ldots ,n,j=1,\ldots ,m\right)$$

where $f_{ij}=r_{ij}/\overset{n}{\underset{i=1}{\sum }}r_{ij}$ . For the applicability of the method, the entire term $f_{ij}\;\cdot \;\ln f_{ij}$ is set to 0 if $f_{ij}=0$ . To give more importance to those indicators that contain more information, the weights $w_{j}$ were computed as defined in Eq. (4).


(4) $$w_{j}=\frac{1-E_{j}}{m-\sum_{j=1}^{m}E_{j}},\overset{m}{\underset{j=1}{\sum }}w_{j}=1,\left(j=1,\;\ldots ,\;m\right)\;$$

#### 2.2.2. Ranking of administrative units

Once the weights were determined, the Technique for Order of Preference by Similarity to Ideal Solution (TOPSIS) (53) was utilized to aggregate them and generate a composite measure of each administrative unit’s contribution to One Health. Here, TOPSIS was used to evaluate the proximity of a set of alternatives (administrative units in this case) to an ideal solution in terms of One Health.

The ideal administrative unit represents a theoretical scenario with the best scores for all the indicators related to the facilitation of zoonotic disease spread. In practice, this scenario is highly unlikely, as it should usually be the case that the highest values of the indicators are distributed over several administrative units. Therefore, TOPSIS calculates the distance between these real solutions and the ideal solution through a series of steps. Again, the first one was to normalize the decision-making matrix. In this case, a vector normalization as shown in Eq. (5) is proposed for the TOPSIS method (54).


(5) $$r_{ij}=\frac{x_{ij}}{\sqrt{\sum_{j=1}^{m\;}x_{ij}^{2}}}$$

The weights $w_{j}$ yielded by Eq. (4) were then multiplied by the normalized values $r_{ij}$ in Eq. (5) to result in a set of normalized weighted values $v_{ij}$ . These were in turn used to determine the positive ( $A^{+}$ ) and negative ( $A^{-}$ ) ideal solutions through Eqs. (6) and (7), respectively.


(6) $$A^{+}=\left\{\left(\max v_{ij}\;|\;j\in J\right),\left(\min v_{ij}\;|\;j\in J^{\prime }\right),\;i=1,2,\ldots ,\;m\right\}=\left\{v_{1}^{+},v_{2}^{+},\ldots ,v_{n}^{+}\right\}$$

(7) $$A^{-}=\left\{\left(\min v_{ij}\;|\;j\in J\right),\left(\max v_{ij}\;|\;j\in J^{\prime }\right),\;i=1,2,\ldots ,\;m\right\}=\left\{v_{1}^{-},v_{2}^{-},\ldots ,v_{n}^{-}\right\}$$

where J and $J^{\prime }$ represent indicators that are beneficial and harmful to the spread of zoonotic diseases, respectively. The distances ( $d_{i}^{+}$ and $d_{i}^{-}$ ) from the actual administrative units to these ideal solutions were calculated by applying Eqs. (8) and (9).


(8) $$d_{i}^{+}=\sqrt{\overset{n}{\underset{j=1}{\sum }}{\left(v_{ij}-v_{j}^{+}\right)}^{2}}$$

(9) $$d_{i}^{-}=\sqrt{\overset{n}{\underset{j=1}{\sum }}{\left(v_{ij}-v_{j}^{-}\right)}^{2}}$$

Finally, the Relative Closeness ( $RC_{i}$ ) from the administrative units to the ideal solution was computed using Eq. (10). The higher the value of $RC_{i}$ , the more susceptible the administrative unit is to zoonoses (the less it contributes to One Health), and vice versa.


(10) $$RC_{i}=\frac{d_{i}^{-}}{d_{i}^{-}+d_{i}^{+}}$$

### 2.3. Correlation between the number of infectious diseases and Green Infrastructure

The validity of the values of $RC_{i}$ obtained from Eq. (10) was assessed by comparing them with the records of infectious disease counts in the case study area. This comparison was conducted using Pearson’s r correlation coefficient (55) since both variables were quantitative (continuous). The presence of a statistically significant association was determined at a significance level ( α ) of 0.05. Therefore, the proposed indicator system was considered valid if it exhibited a strong positive correlation with the infectious disease count, and the value of *p* obtained from Pearson’s r was less than α .

Moreover, the results were also analyzed in terms of their relationship with Green Infrastructure (GI). The potential benefits of GI for the three pillars of One Health have been discussed in previous literature (24). GI can be defined as “a strategically planned network of natural and semi-natural areas with other environmental features designed and managed to deliver a wide range of ecosystem services. It incorporates green spaces (or blue if aquatic ecosystems are concerned) and other physical features in terrestrial (including coastal) and marine areas” (56).

The correlation between One Health and GI was determined by the correlation coefficient between these values of $RC_{i}$ and the area covered by GI in each administrative unit. The latter was determined based on the following classes in the Corine Land Cover (CLC) (57): 1.4 (artificial, non-agricultural vegetated areas), 2 (agricultural areas), 3.1 (forest), 3.2 (shrub and/or herbaceous vegetation associations), 4 (wetlands), and 5.1 (inland waters). These categories allowed for differentiation between artificial (class 1.4) and natural and semi-natural (classes 2, 3.1, 3.2, 4, and 5) GI.

## 3. Results and discussion: a case study in the Valencian Community (Spain)

**Figure 2:**
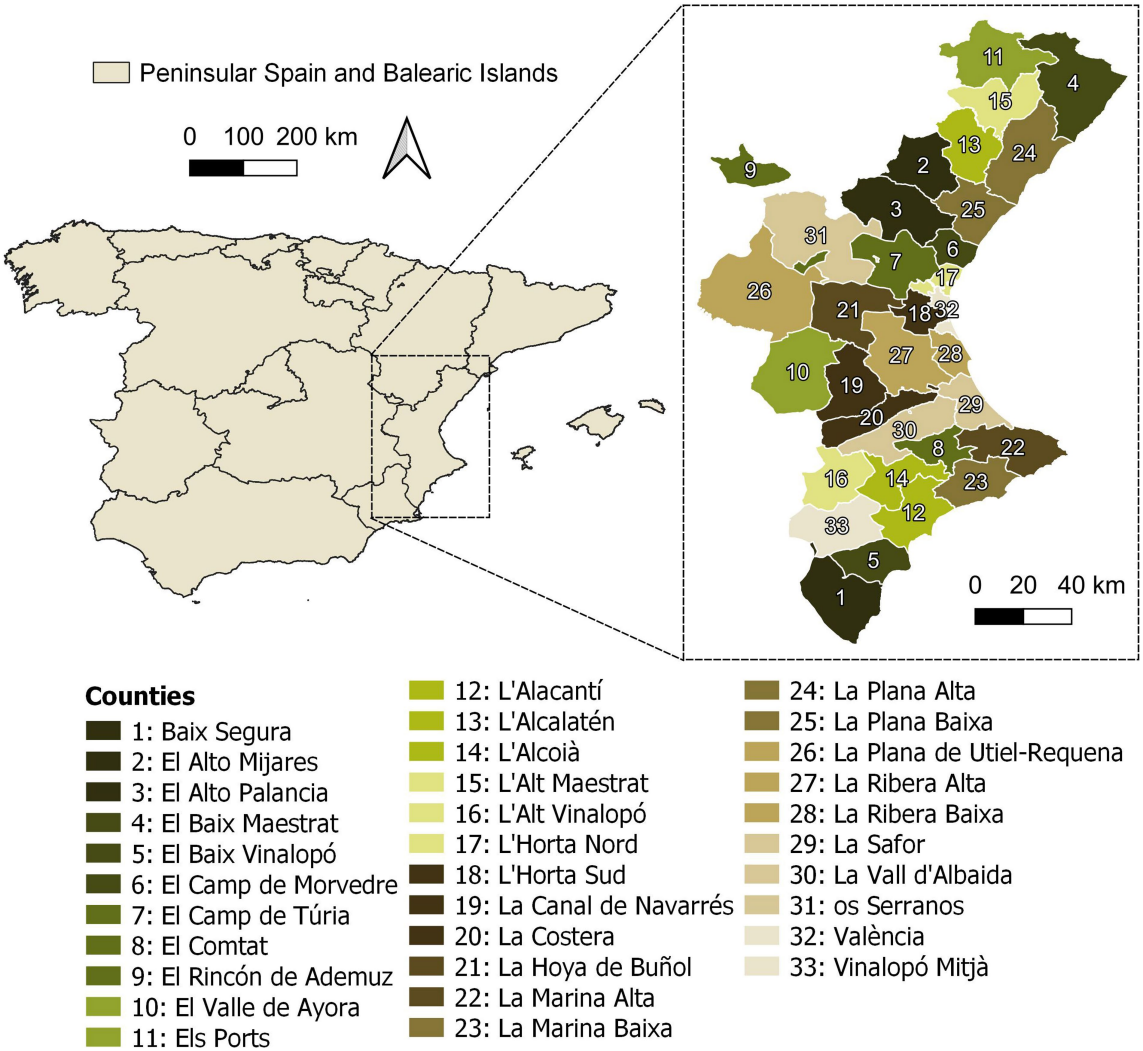
Situation map of the Valencian community and geographical extent of its counties.

The indicator system was tested in the 33 counties of the Valencian Community as shown in Figure 2. The Valencian Community is located in eastern Spain and has a predominantly Mediterranean, arid, and semi-arid climate. Regions with Mediterranean ecosystems, like the Valencian Community, are known to experience high levels of environmental degradation due to biophysical factors such as wildfires, drought, erosion, as well as social factors such as tourism, urbanization, and deforestation (58, 59).

In terms of biophysical factors, it is noteworthy that 17% of the forest area in the region has experienced at least one wildfire, 29% suffers from serious erosion problems, and 46% is at risk of desertification. In addition, the effects of climate change significantly affect the regularity of precipitation, which can have implications for areas prone to desertification (58, 60).

Concerning social factors, the region is characterized by a dominance of the service sector, which accounts for more than 65% of total employment, and tourism activities, such as real estate, gastronomy, and transportation, which have an employment rate of over 12% and contribute 15% to the GDP (61, 62). In particular, the Valencian Community has cultivated a tourism model focused on second homes linked to construction that has resulted in serious environmental impacts, high space requirements, and an unsustainable approach (63).

All these activities have been boosted by lax political guidelines and favorable economic incentives, leading to land degradation (58) (EVR, 2020). Thus, insufficient budgetary resources further contribute to the challenge of meeting the goal of recovering 15% of degraded ecosystems. Besides, other studies indicate that the Valencian Community has primarily focused on reforestation projects with limited impact on biodiversity, while neglecting medium and long-term-ecological restoration projects (64, 65).

### 3.1. Selection of indicators

**Figure 3:**
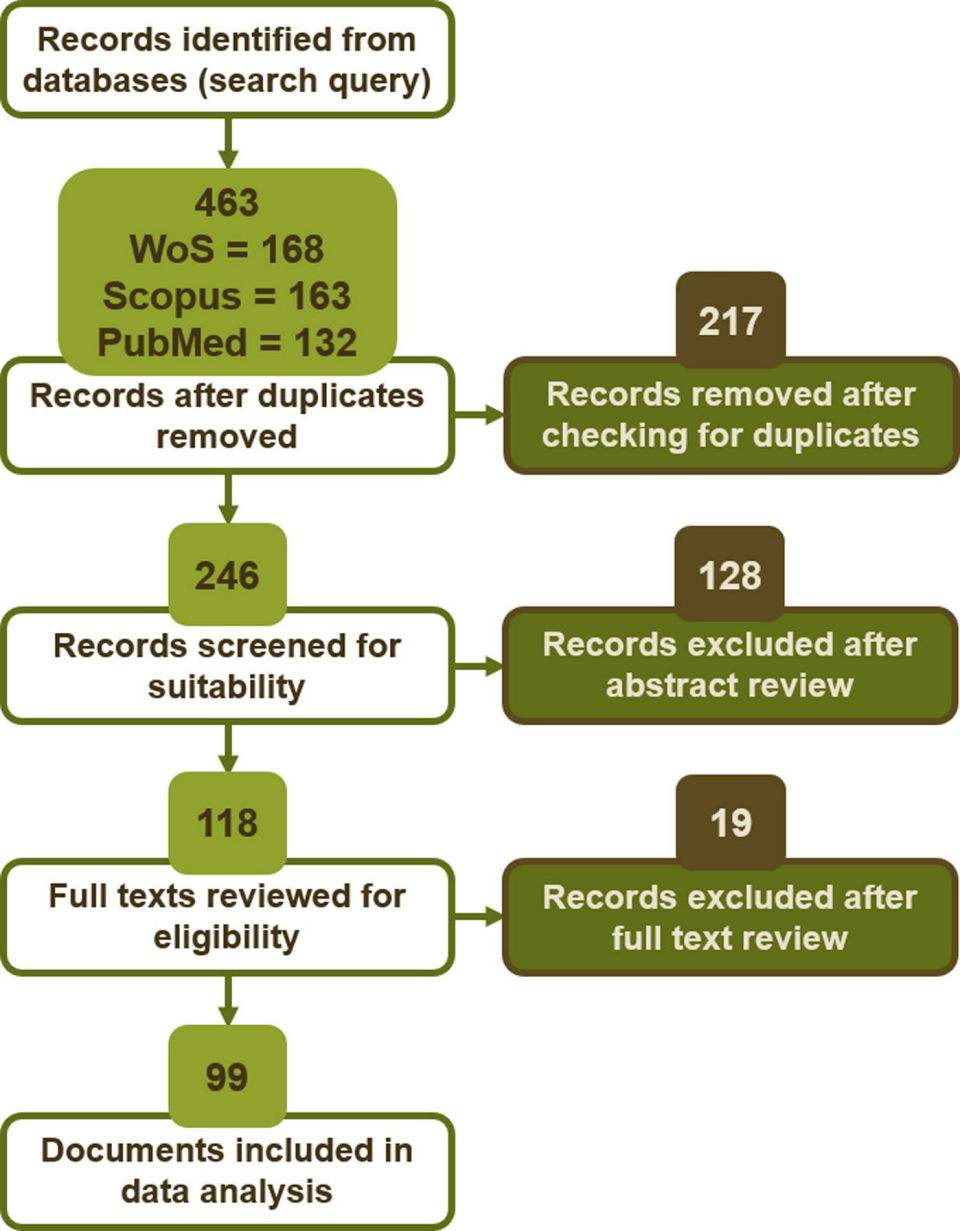
Number of records retained and removed after each step of the systematic literature review.

The systematic literature review conducted following the PRISMA statement produced the results presented in Figure 3, which shows the number of records removed at each step. The search query formulated in Eq. (1) initially returned 463 records from the three databases considered. This number gradually decreased through the different steps of the PRISMA statement until a final number of 99 articles was obtained for data analysis.

The extraction of information from these articles allowed for the identification of several indicators that could potentially influence the spread of diseases impacting human, animal, and environmental health. The vast majority of these indicators were not used as direct metrics to model spatial facilitation of disease spread but were instead discussed as factors that may affect One Health. Table 1 compiles the list of indicators extracted from the review and provides the main references that supported their use as surrogates for aspects that go against the One Health concept (9, 14, 40, 66–161).

**Table 1:** List of proposed or derivable indicators from the systematic literature review on metrics used to address One Health.

Indicators proposed or derivable	Reference(s)
Crops; Agriculture	(66–68)
Hunting spaces, human-wildlife interface; and crops-animals-water interface	(69–71)
Exposure of farm animals to water pollution	(72–74)
Income *per capita*; access to water supply	(40, 75)
Wildlife population	(76, 77)
Soil pollution	(78, 79)
Cardiovascular diseases; diabetes	(80)
Poverty; livestock movement; and population density	(75, 81, 82)
Climate change; extreme weather, healthcare facilities; and education	(83, 84)
Hiking trails; parks	(85)
Seabirds	(86–88)
Social inequalities	(70)
Domestic animals	(89, 90)
Animal bites	(91)
Habitat overlap; wildlife-livestock interactions	(92)
Deforestation	(75, 93)
Bird migration	(71)
Dog abandonment	(94, 95)
Sewage water	(96, 97)
Rainfall, temperature; humidity; and air pollution	(98–101)
Farm outbreaks; rural areas	(102)
Human-livestock interface	(103–105)
Human-bird interface	(106)
Insects-farms interface	(107)
None (at least in the terms that can be useful for this study)	(9, 14, 72, 108–161)

Certainindicators were discarded due to limited data availability, low frequency, and/or the impossibility to characterize them spatially. Based on the frequency of the indicators found to be valid, the list in Table 2 was compiled for subsequent calculations. The first subset of indicators (from $I_{1}$ to $I_{8}$ ) focused on population demographics (either human or animal) and socioeconomic aspects such as health facility density, education level, and financial resources. Another group consisted of indicators related to land use (from $I_{9}$ to $I_{13}$ ). The interactions between animal and human populations and water bodies were addressed in the indicators ranging from $I_{13}$ to $I_{18}$ . The final group of indicators pertained to environmental conditions, including air quality ( $I_{19}$ and $I_{20}$ ) and climate ( $I_{21}$ and $I_{22}$ ).

**Table 2:** List of indicators to model spatial susceptibility to zoonotic diseases.

ID	Indicator	Type	Format
$I_{1}$	Density of health centers (no./km^2^)	Cost	Point
$I_{2}$	Density of veterinary centers (no./km^2^)	Cost	Point
$I_{3}$	Human population density (no./km^2^)	Benefit	Value
$I_{4}$	Share of illiterate population (%)	Benefit	Value
$I_{5}$	Average income per consumption unit (€)	Cost	Value
$I_{6}$	Density of domestic animals (/km^2^)	Benefit	Value
$I_{7}$	Density of farm animals (no./km^2^)	Benefit	Value
$I_{8}$	Density of wild animals (no./km^2^)	Benefit	Point
$I_{9}$	Agricultural land (%)	Benefit	Polygon
$I_{10}$	Deforestation (%)	Benefit	Polygon
$I_{11}$	Livestock (%)	Benefit	Polygon
$I_{12}$	Hunting territory (%)	Benefit	Polygon
$I_{13}$	Water and soil pollution (score)	Benefit	Polygon
$I_{14}$	Humans * Wild animals (score)	Benefit	Polygon/Point
$I_{15}$	Humans * Livestock (score)	Benefit	Polygon
$I_{16}$	Wild animals * Livestock (score)	Benefit	Point/Polygon
$I_{17}$	Wild animals * Water bodies (score)	Benefit	Point/Polygon
$I_{18}$	Livestock * Water bodies (score)	Benefit	Polygon
$I_{19}$	Average concentration of NOx (μg/m^3^)	Benefit	Grid polygon
$I_{20}$	Average concentration of POD6 wheat (mmol/m^2^)	Benefit	Grid polygon
$I_{21}$	Total precipitation (mm)	Benefit	Raster
$I_{22}$	Mean temperature (° C)	Benefit	Raster

The rationale behind the influence of these indicators on One Health is outlined below. The impact of Antimicrobial Resistance (AMR) and its implications for One Health has been linked to reduced accessibility to adequate healthcare and human health ( $I_{1}$ ) as well as veterinary ( $I_{2}$ ) professionalism (83). Moreover, high population densities ( $I_{3}$ , $I_{6}$ , $I_{7}$ and $I_{8}$ ) can contributeto the spread of zoonotic diseases and increased intra-interactions within and between populations and ecosystems (81). In the case of human populations, factors such as poverty ( $I_{5}$ ) can support the evolution of pathogen life cycles, while education ( $I_{4}$ ) can aid in controlling global zoonotic pathogens (75).

The impact of agriculture ( $I_{9}$ ) is associated with freshwater, which serves as a common drinking source for wildlife (69), as reflected in $I_{17}$ . Agricultural runoff can be a source of pollution, especially if antibiotics are utilized in farming practices (68). The use of agrochemicals in agriculture contributes to reduced biodiversity and promotes mutation among microbial populations in soil and water bodies ( $I_{13}$ ) (162). Other forms of anthropogenic land use changes such as deforestation ( $I_{10}$ ) are linked to the increased adaptability of disease vectors and the creation of the conditions for increased interactions at the wildlife-human interface (163). $I_{11}$ and $I_{12}$ relate to aspects like the role of hunted animals as carriers of bacteria with specific resistance traits (92) and livestock movement as a contributing factor to the emergence of zoonotic diseases (164).

Increased global connectivity between humans and animals (either livestock or wildlife; $I_{14}$ and $I_{16}$ ) has been identifiedas a source of acceleratedand exacerbated AMR (70). Surface water (streams, rivers, lakes, and ponds) used as drinking water for dairy cattle can serve as a source of pathogens (72) ( $I_{18}$ ), posingbilateral implications for farm animals and humans ( $I_{15}$ ) due to the dissemination and maintenance of resistance genes in the environment (165).

The impact of air pollution ( $I_{19}$ and $I_{20}$ ) is indirect as it stems from its contribution to biodiversity decline (166), which in turn has been argued to increase human exposure to zoonotic pathogens (167). Moisture resulting from precipitation ( $I_{21}$ ) results in dense vegetation that provides suitable conditions for vector proliferation (168). Floods caused by heavy rainfall also increase the risk of waterborne diseases (169). Temperature ( $I_{22}$ ) is proportional to vector distribution and disease risk too. High temperatures promote increased activity of mosquitoes, ticks, and sandflies, while they can lead to the migration of rodents into human habitats (170, 171).

### 3.2. Processing of indicators

Some indicators in Table 2 were obtained directly as single values per administrative unit, while others were available as either vector (point or polygon) or raster layers. These data had to be processed to express them as a single value per administrative unit. The indicators in point format were processed to obtain densities per county. Instead, polygon data were used to determine the proportion of the area corresponding to the indicators covered by the counties.

The indicators related to the interactions between point and polygon layers ( $I_{14}$ , $I_{16},$ and $I_{17}$ ) were determined through a three-step process: transform the point layer into a raster using a Kernel density function (shape = quartic, radius = 25 km), calculate the interaction of the gridded density and the polygon layer by multiplication, and aggregate the values per county by taking the median of the interaction values per raster unit into a polygon layer.

Apart from these general procedures, two indicators required specific calculations. Deforestation ( $I_{10}$ ) was determined based on the variations in class 3.1 (Forests) in the 2006, 2012, and 2018 CLC maps in the Valencian Community. The CLC map was also used to characterize water and soil pollution ( $I_{13}$ ), which was computed by assigning scores depending on the land cover classes (172).

**Figure 4:**
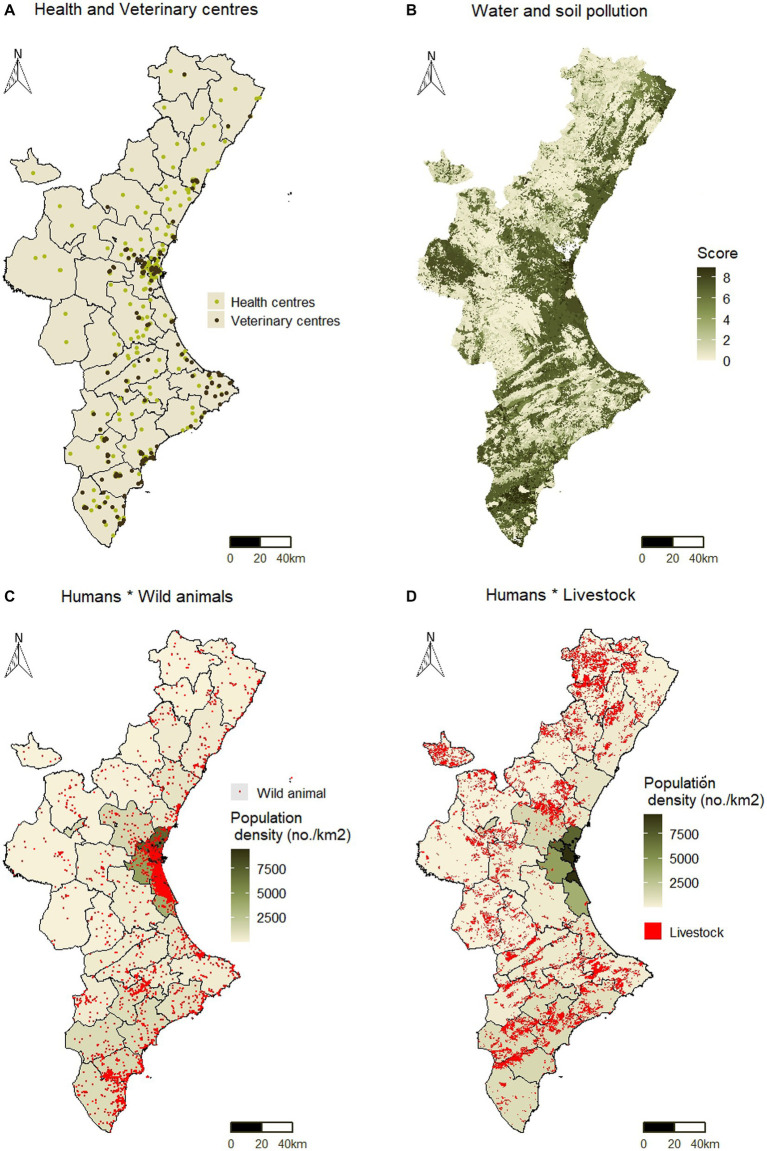
Processing of indicators before their calculation per county **(A)** Health and veterinary centers ( $I_{1}$ and $I_{2}$ ); **(B)** Water and soil pollution ( $I_{13}$ ); **(C)** Interaction between human population and wildlife ( $I_{14}$ ); and **(D)** Interaction between human population and livestock ( $I_{15}$ ).

Figure 4 shows various steps in the processing of indicators as an example. Figure 4A depicts the location of health and veterinary centers in the study area, which were used to obtain their density per county ( $I_{1}$ and $I_{2}$ ). Figure 4B applies the aforementioned scores for water and soil pollution to the different land covers in the study area. Figures 4C,D represent the presence of wild animals and livestock areas in the region along with the human population values per county, which were the inputs used to characterize $I_{14}$ and $I_{15}$ . The values per county for each indicator considered ( $I_{1}$ - $I_{22}$ ) are provided as Supplementary material.

#### 3.2.1. Weighting of indicators

**Figure 5:**
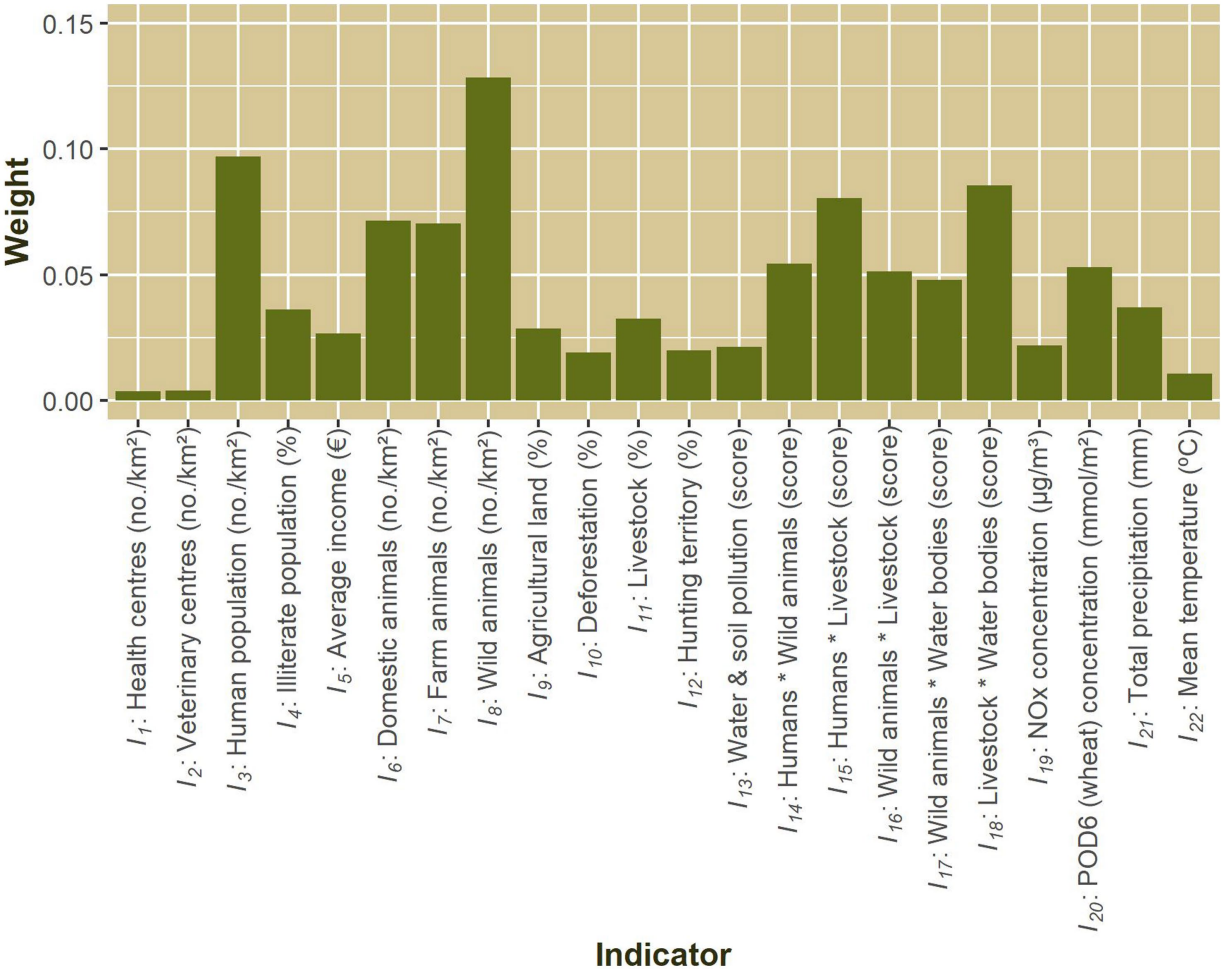
Weights obtained for the proposed indicators using the Entropy Method (EM).

The application of the EM algorithm according to Eqs. (2)–(4) resulted in the weights shown in Figure 5. These weights indicate the importance of various indicators for this study. Some indicators were found to have reduced importance (weights less than 0.025 out of 1): including healthcare facilities (for humans or animals), temperature, and NOx concentration, deforestation, hunting territory, and water and soil pollution. According to the EM principle, these variables are not sufficiently discriminatory among the counties in the Valencian Community, indicating that most counties are relatively homogeneous in relation to these indicators.

Instead, the indicators related to human and animal populations and their interactions obtained the highest weights. In particular, the presence of wildlife and humans was identified as the two most important indicators. This highlights the significance of distinct habitats and the risks associated with phenomena such as urban sprawl. The next most important indicators were also aligned with this focus, involving domestic and farm animals and their interactions with wildlife, humans, and water bodies, with the latter also being represented by precipitation. The ozone uptake by wheat was also identified as an important environmental indicator due to its impact on food safety.

Apart from the rationale behind them, all of these indicators with the highest weights exhibited spatial variability among the counties in the study area. In addition, they accounted for the three dimensions of One Health. Among them, animal-related variables were the most important ( $I_{6}$ - $I_{8}$ , $I_{14}$ - $I_{18}$ ), followed by those focused on humans ( $I_{3}$ , $I_{14}$ , and $I_{15}$ ) and the environment ( $I_{17}$ , $I_{20},$ and $I_{21}$ ).

#### 3.2.2. Ranking of administrative units

**Figure 6:**
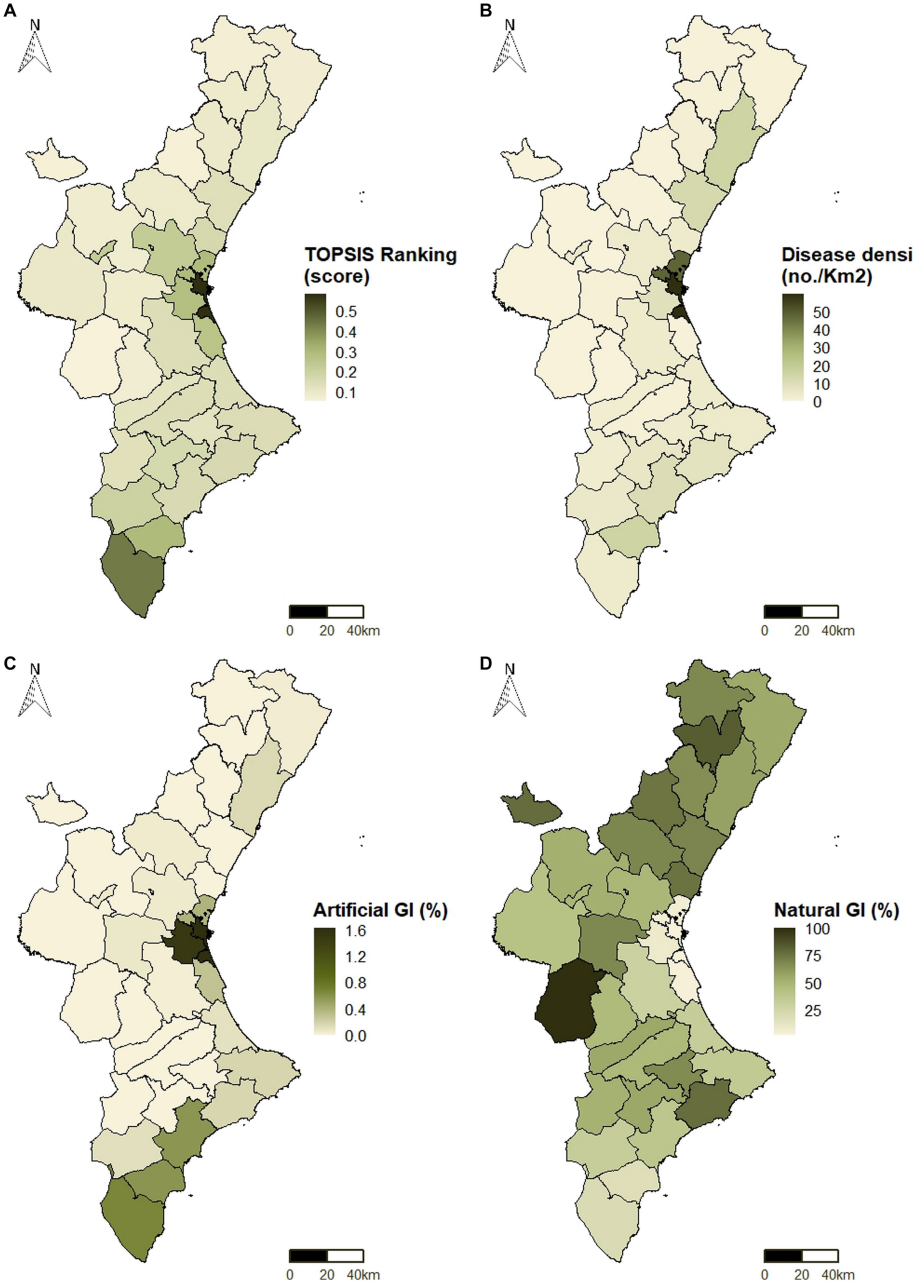
**(A)** Susceptibility to zoonoses according to the indicator system; **(B)** Infectious disease density (cases/km^2^); **(C)** Proportion of artificial GI (%); and **(D)** Proportion of natural and semi-natural GI (%).

The use of Eqs. (5)–(10) following the steps of the TOPSIS method resulted in the map shown in Figure 6A. The results are consistent with the significance of indicators associated with humans and wildlife (Figure 5), as well as the distribution of these populations (Figure 4A). According to this map, the primary focus of disease spread is observed in the county of València (Figure 2), where the region’s capital is situated, highlighting the implications of population movement in this area.

The second county in the ranking of zoonotic disease susceptibility was el Baix Segura, located in the southern of the Valencian Community. Although the human population density is not as high in this county, it attained the highest scores in terms of wildlife-livestock-water interactions. Instead, counties in the western and northern regions exhibited lower susceptibility. As can be seen from the results provided as Supplementary material, these counties only obtained high scores in indicators of lesser importance (Figure 5), such as illiterate population ( $I_{4}$ ), average income per consumption unit ( $I_{5}$ ), or hunting territory ( $I_{12}$ ).

Overall, the results presented in Figure 6A are instructive in terms of indicating hotspots linked to susceptibility to infectious diseases. The indicator system based on the EM and TOPSIS methods enables the derivation of discriminative results, highlighting substantial differences between the most critical counties and the others. Consequently, this facilitates the implementation of strategies to strengthen One Health through measures aimed at safeguarding the interface between humans, animals, and the environment.

### 3.3. Correlation between the number of infectious diseases and green infrastructure

The Valencian Animal Identification Registry (RIVIA in Spanish) provides a report on the cases notified in 2021 for the following diseases: Leishmaniosis, Ehrlichiosis, Dirofilariosis, Leptospirosis, Toxoplasmosis, and Babesiosis. Figure 6B shows how these cases are distributed across the counties in the Valencian Community.

**Figure 7:**
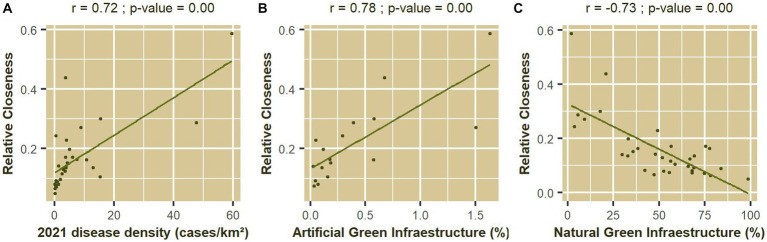
Correlation coefficient between the results of applying the indicator system to the Valencian Community and **(A)** Infectious disease density reported in 2021 (cases/km^2^); **(B)** Proportion of artificial GI (%); and **(C)** Proportion of natural and semi-natural GI (%).

Figure 7A demonstrates that the value of Pearson’s r obtained between disease density (number of records per county area) and the values of $RC_{i}$ was 0.72 (value of *p* < 0.05). This strong and positive correlation coefficient reinforces the effectiveness of the proposed indicator system in representing a region’s susceptibility to the emergence of infectious diseases.

A similar analysis was carried out to examine the relationship between $RC_{i}$ and the proportion of artificial (Figure 6C) and natural and semi-natural GI (Figure 6D) in the study area. Again, the *p* values were below the significance level of 0.05 in both cases, while the values of Pearson’s r obtained were 0.78 (Figure 7B) and − 0.73 (Figure 7C), respectively. These results indicate that more developed and urbanized counties exhibit a higher susceptibility to the emergence of infectious diseases, while the presence of natural and semi-natural areas may contribute to the One Health initiative.

## 4. Discussion

The results obtained in this study address some key demands in the field of landscape epidemiology, including the incorporation of spatial interactions between individuals and environmental gradients in large-scale studies (173). Spatial dimensions such as distances between humans, animals, and environments have been found to be associated with both directly and indirectly transmitted infectious diseases (174). Overall, the spatial processing of indicators proposed in this study aligns with these premises.

The results are also consistent with the notion that urbanization contributes to increased encounters with wildlife, leading to challenges in infectious disease epidemiology due to amplified and faster spread (175). Although the interface between human and wildlife populations ( $I_{14}$ ) carries moderate weight, wild animals ( $I_{8}$ ) and human population ( $I_{3}$ ) were identified as the two most important indicators according to Figure 5.

Other authors have emphasized that land urbanization, rather than population urbanization is a key driver of infectious disease morbidity and mortality (176). This does not necessarily contradict the results obtained in this study but underscores the importance of using appropriate metrics. Population density goes beyond population size by considering how the accumulation of people and animals can facilitate disease transmission.

Urbanization promotes the occurrence of zoonoses through demographic growth and density, socioeconomic inequalities, increased movement of people and animals, and land use change (177). All these factors are included in the list of indicators presented in Table 1. The results in Figure 7 would be supported by this line of thought, since considering these aspects together correlates positively with the number of infectious diseases and the presence of natural and semi-natural GI, while correlating negatively with artificial GI in more urbanized areas.

The role of GI is linked to biodiversity, which is a crucial factor to consider when pursuing One Health goals. Zoonotic pathogens are more likely to originate from specific taxa that often reproduce due to human influences, i.e., in urbanized areas lacking biodiversity values (167). This may explain the lower susceptibility to infectious diseases in counties with higher proportions of natural and semi-natural GI. Notably, the provision of suitable habitats for vector and zoonotic reservoir populations is one of the regulating ecosystem services supported by GI (178).

Therefore, the results of the indicator system can support the development of planning strategies aimed at promoting the principles of One Health. Strategies could focus on the implementation of natural GI, as the presence of these areas showed a negative correlation with zoonoses. Preserving and/or restoring natural landscape features can help minimize habitat encroachment, improve land and water quality, and mitigate extreme atmospheric conditions. Instead, artificial green spaces, commonly found in urban areas, showed a positive correlation with zoonoses. This is associated with urban sprawl and its effects on increased interactions between populations through greater movement of people, animals, and wildlife between developed and undeveloped areas.

Although these conclusions stem from validated results, this investigation had some limitations that constrain its impact. First, the data used to characterize certain indicators were site-specific, which hampers the usability of the indicator system in other parts of the world where such data may be lacking. Second, the methodology behind the indicator system could benefit from automation, which would result in a web-based application where users only need to input the data to obtain the composite metric of zoonotic disease susceptibility, while calculating the GIS and MCDA tasks would be in the background. Finally, this indicator system may have limitations in modeling diseases transmitted by vectors such as ticks or mosquitoes, whose distribution and survival are influenced by complex dynamics determined by climatic and seasonal factors, as well as by the presence of specific hosts, with certain conditions being favorable for some species but not for others (179, 180).

## 5. Summary and concluding remarks

This research consisted of the development, application, and validation of an indicator system to model spatial susceptibility to zoonotic disease facilitation, thus providing an indirect measurement of contributions to the One Health initiative. The study focused on the counties of the Valencian Community (Spain) as a case study. The methods used to achieve this goal included a systematic literature review, the combination of GIS and MCDM techniques, and the use of statistical testing.

The systematic literature review resulted in the identification of 22 spatial indicators that encompassed population, land use, and atmospheric variables. These indicators represented the risks associated with direct or indirect interactions at the interfaces between humans, wildlife, livestock, and ecosystems, thereby addressing the three pillars of the One Health approach: human, animal, and environmental health.

Processing these indicators using GIS and MCDM methods resulted in a composite metric for zoonotic disease susceptibility. The results showed that the indicators concerning human and animal populations and their interactions are the most important ones, underlining the relevance of controlling urban sprawl to mitigate habitat encroachment. The impact of urbanization was also further supported by the county level analysis, with the highest susceptibility to zoonotic diseases observed in the county corresponding to the capital and most populated city in the region.

This trend was confirmed by the strong positive correlation between the results of the indicator system and the presence of artificial GI. Conversely, the association with natural and semi-natural GI exhibited an opposite relationship, underscoring the importance of these areas for habitat preservation and biodiversity protection, thus aligning with the principles of One Health. The validity of these findings was verified by comparing the results of the indicator system with the records reported in the Valencian Community in 2021, showing a strong positive correlation. Therefore, the indicator system is proposed as a tool for implementing the principles of the One Health approach when designing strategies for better public space planning.

## Data availability statement

The original contributions presented in the study are included in the article/Supplementary material; further inquiries can be directed to the corresponding author.

## Author contributions

DJ-E conceived the research, contributed to the literature review, analyzed the data, discussed the results, and wrote the manuscript. FM-V contributed to the literature review, produced the results, and wrote the manuscript. VM contributed to the literature review and produced the results. FC-R contributed to the literature review, wrote the manuscript, and reviewed the manuscript. LB contributed to the literature review and reviewed the manuscript. All authors contributed to the article and approved the submitted version.

## Funding

This study was financed through the research projects ECOVAL (grant number CIGE/2021/079) and ECOHEALTH (grant number PII2022_04), funded by the Conselleria for Innovation, Universities, Science and Digital Society of the Generalitat Valenciana and the Valencian International University (VIU), respectively.

## Conflict of interest

The authors declare that the research was conducted in the absence of any commercial or financial relationships that could be construed as a potential conflict of interest.

## Publisher’s note

All claims expressed in this article are solely those of the authors and do not necessarily represent those of their affiliated organizations, or those of the publisher, the editors and the reviewers. Any product that may be evaluated in this article, or claim that may be made by its manufacturer, is not guaranteed or endorsed by the publisher.
