# Supplementary figures and images for: Toward One Health: a spatial indicator system to model the facilitation of the spread of zoonotic diseases
Source: Front Public Health. 2023 Jun 29;11:1215574. doi: 10.3389/fpubh.2023.1215574 (PMC10340543; doi:10.3389/fpubh.2023.1215574)

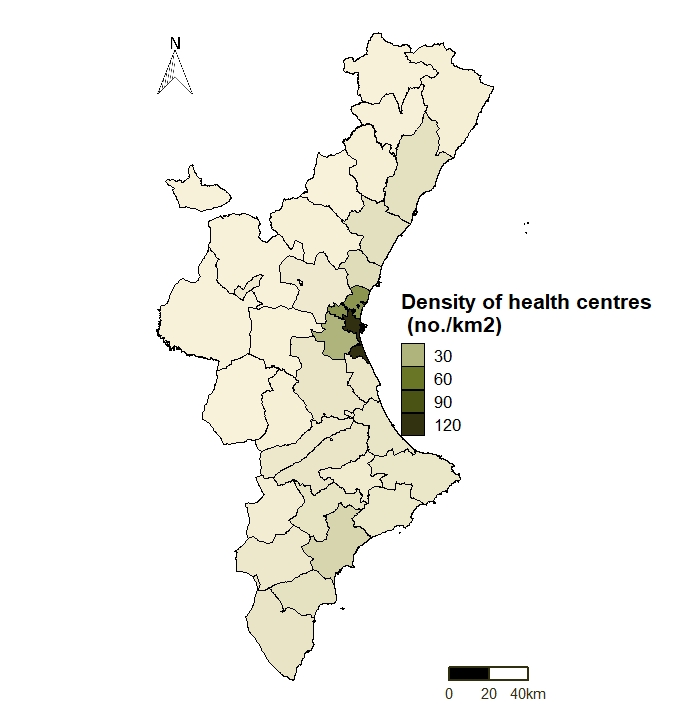

Supplement: Supplementary file 1 [file Data_Sheet_1.ZIP › I1.jpeg]

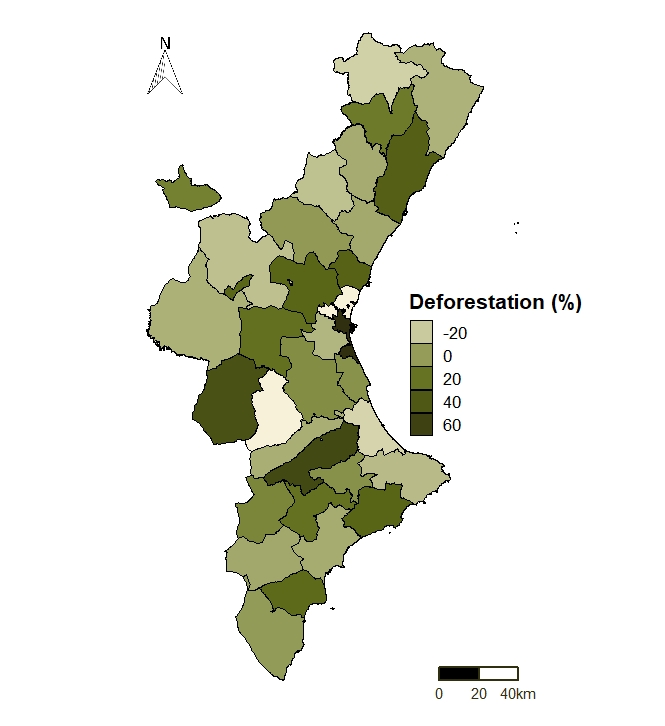

Supplement: Supplementary file 1 [file Data_Sheet_1.ZIP › i10.jpeg]

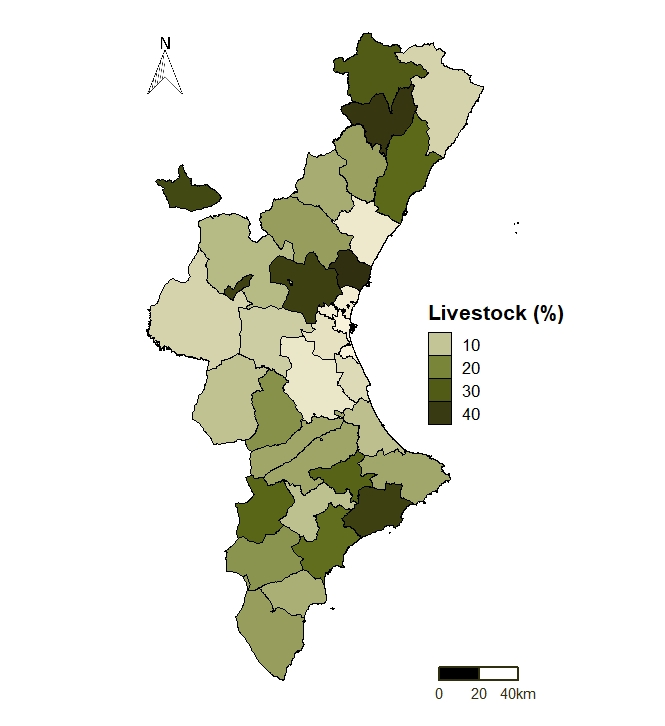

Supplement: Supplementary file 1 [file Data_Sheet_1.ZIP › i11.jpeg]

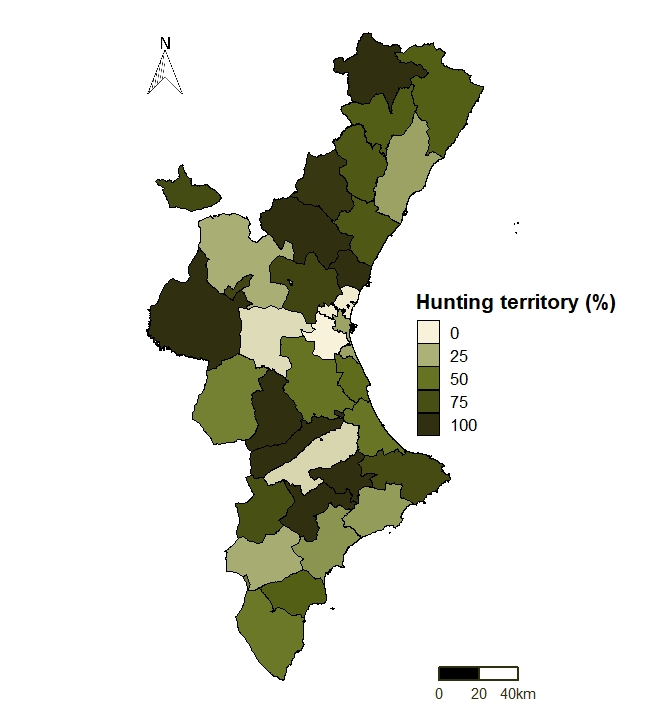

Supplement: Supplementary file 1 [file Data_Sheet_1.ZIP › i12.jpeg]

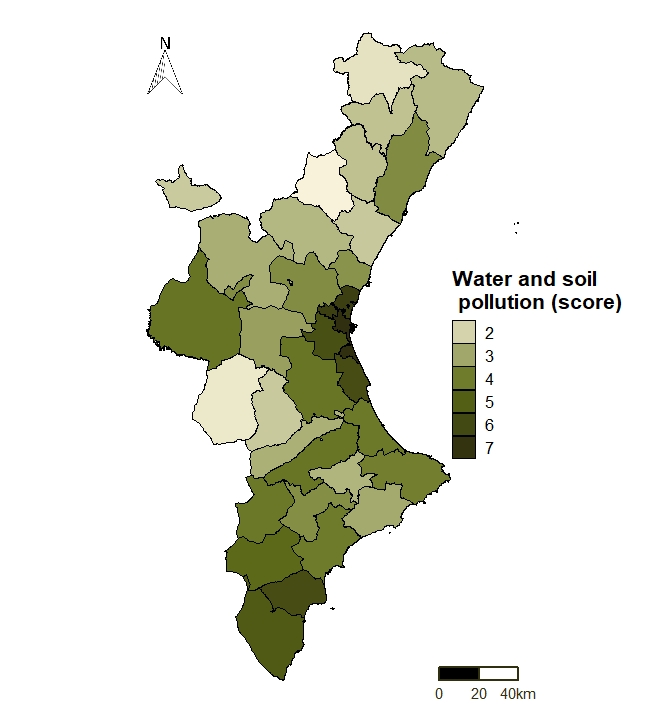

Supplement: Supplementary file 1 [file Data_Sheet_1.ZIP › i13.jpeg]

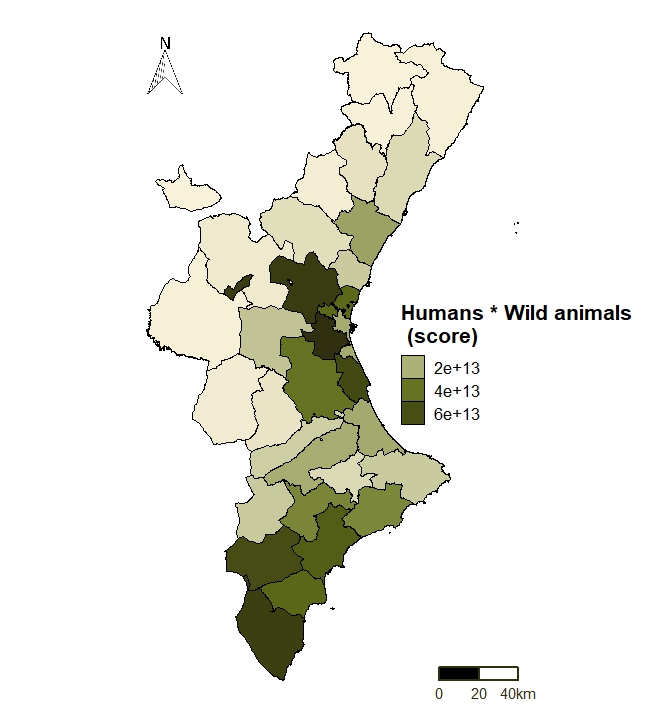

Supplement: Supplementary file 1 [file Data_Sheet_1.ZIP › i14.jpeg]

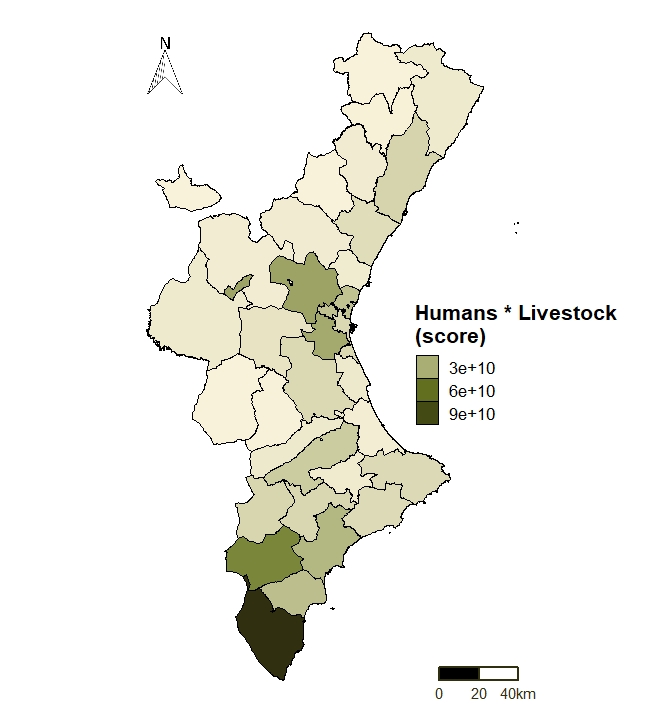

Supplement: Supplementary file 1 [file Data_Sheet_1.ZIP › i15.jpeg]

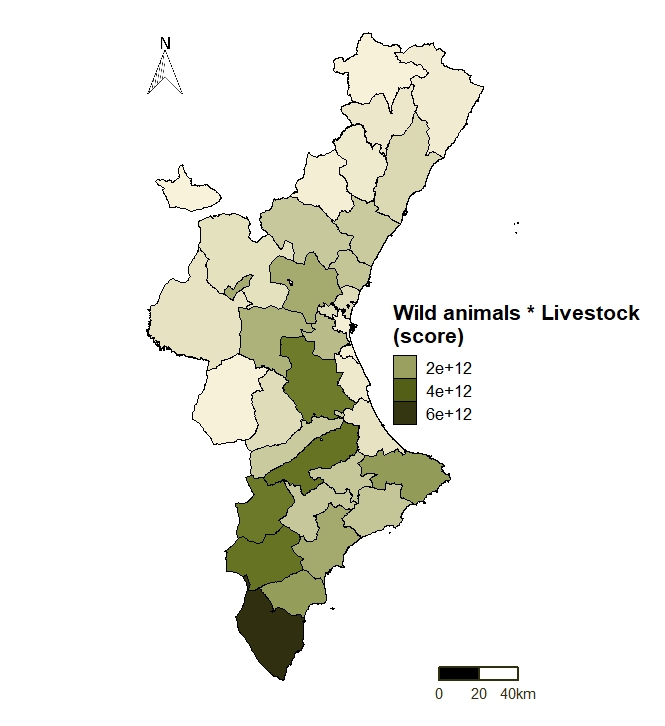

Supplement: Supplementary file 1 [file Data_Sheet_1.ZIP › i16.jpeg]

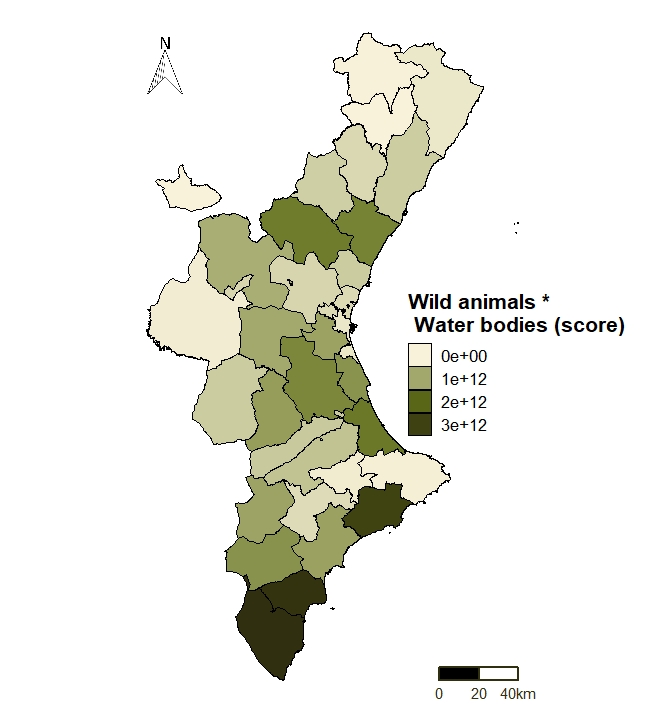

Supplement: Supplementary file 1 [file Data_Sheet_1.ZIP › i17.jpeg]

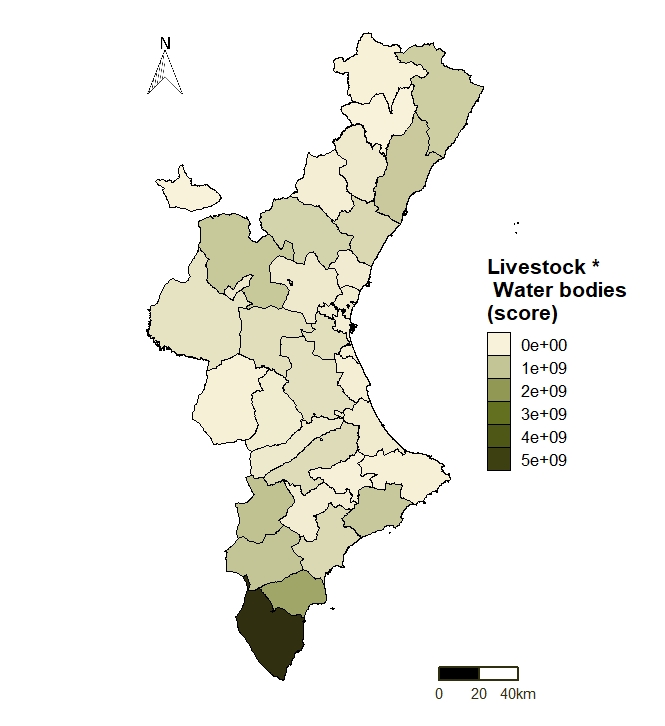

Supplement: Supplementary file 1 [file Data_Sheet_1.ZIP › i18.jpeg]

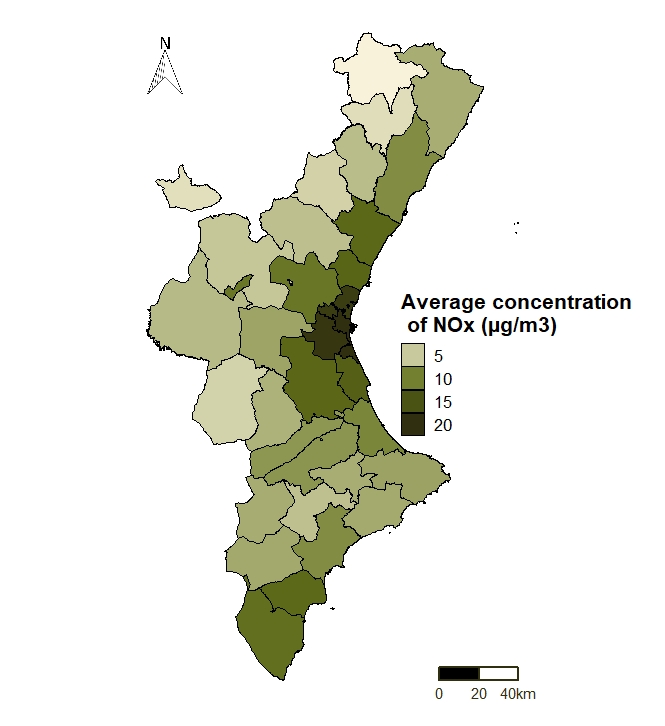

Supplement: Supplementary file 1 [file Data_Sheet_1.ZIP › i19.jpeg]

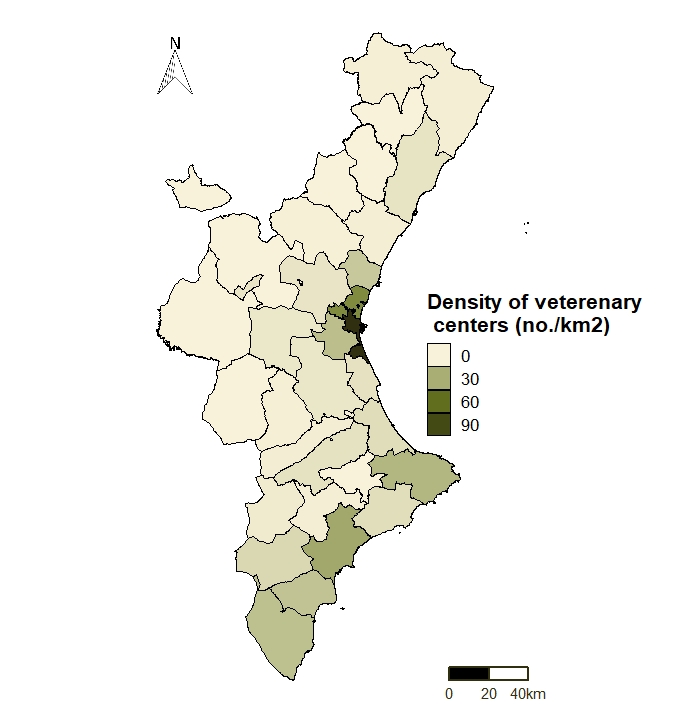

Supplement: Supplementary file 1 [file Data_Sheet_1.ZIP › i2.jpeg]

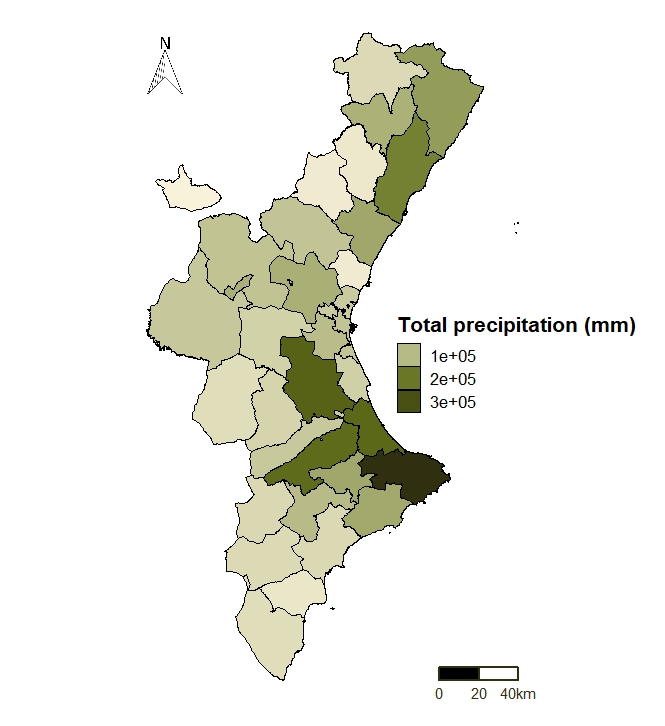

Supplement: Supplementary file 1 [file Data_Sheet_1.ZIP › i21.jpeg]

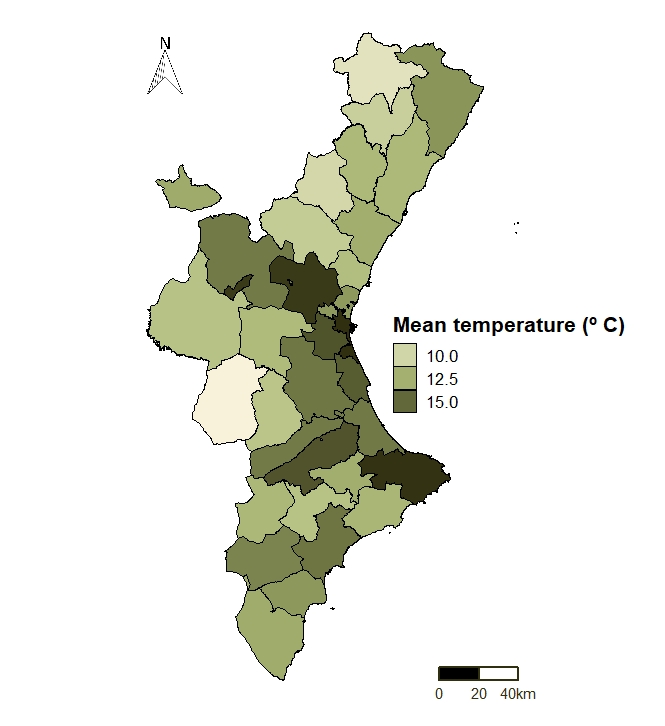

Supplement: Supplementary file 1 [file Data_Sheet_1.ZIP › i22.jpeg]

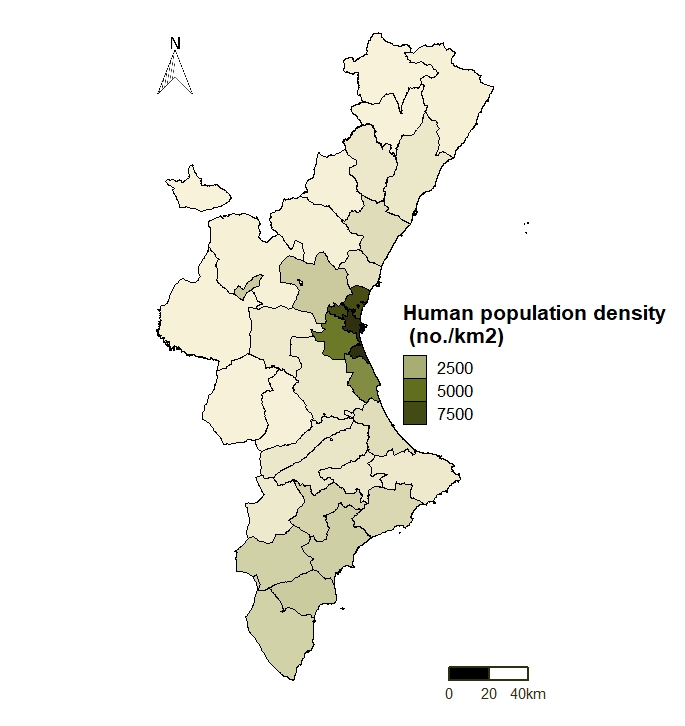

Supplement: Supplementary file 1 [file Data_Sheet_1.ZIP › i3.jpeg]

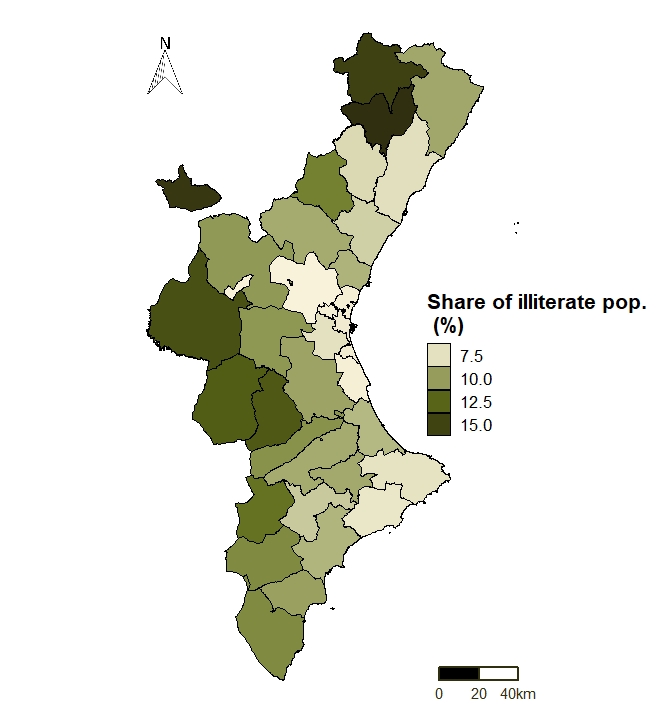

Supplement: Supplementary file 1 [file Data_Sheet_1.ZIP › i4.jpeg]

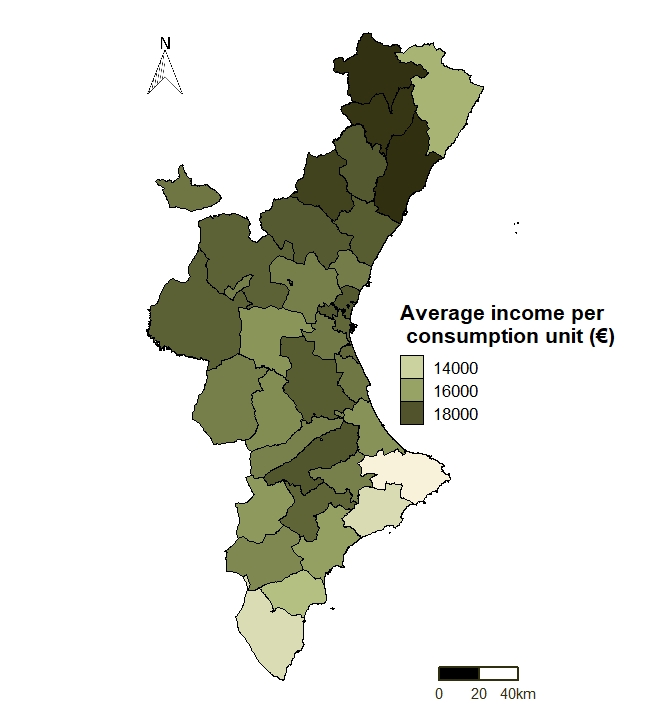

Supplement: Supplementary file 1 [file Data_Sheet_1.ZIP › i5.jpeg]

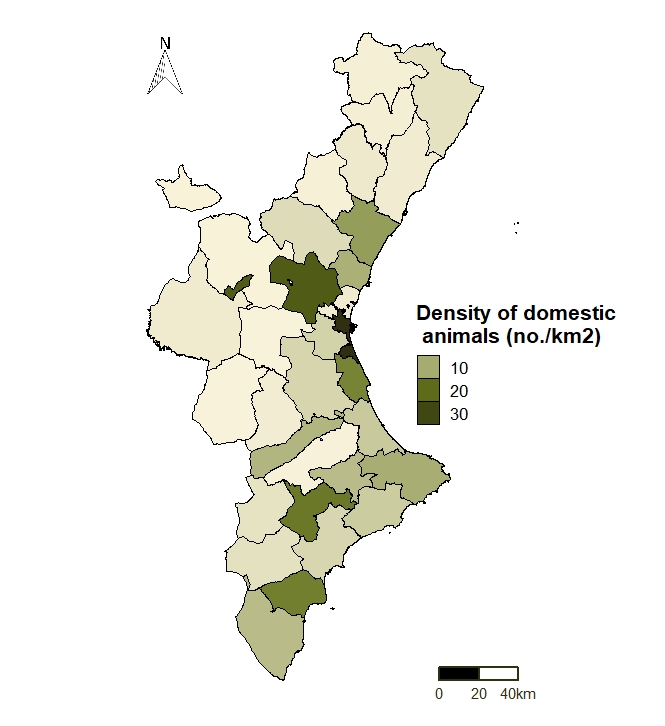

Supplement: Supplementary file 1 [file Data_Sheet_1.ZIP › i6.jpeg]

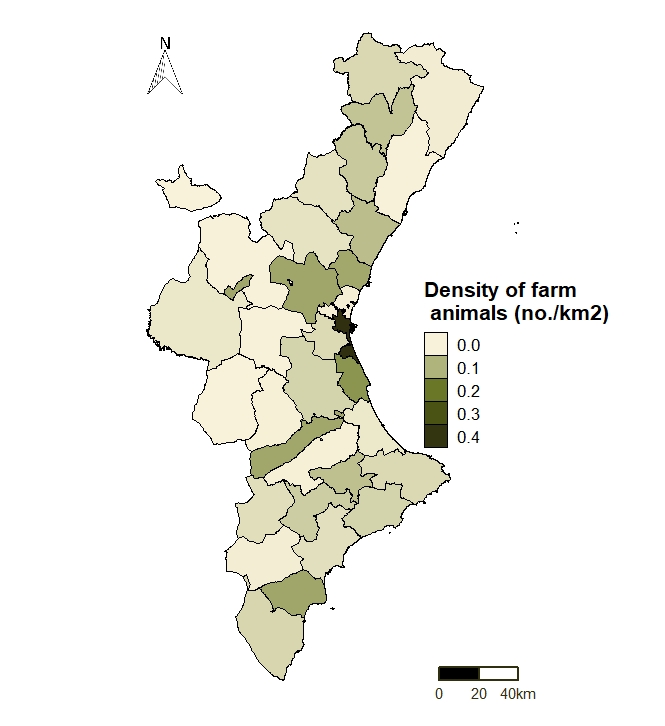

Supplement: Supplementary file 1 [file Data_Sheet_1.ZIP › i7.jpeg]

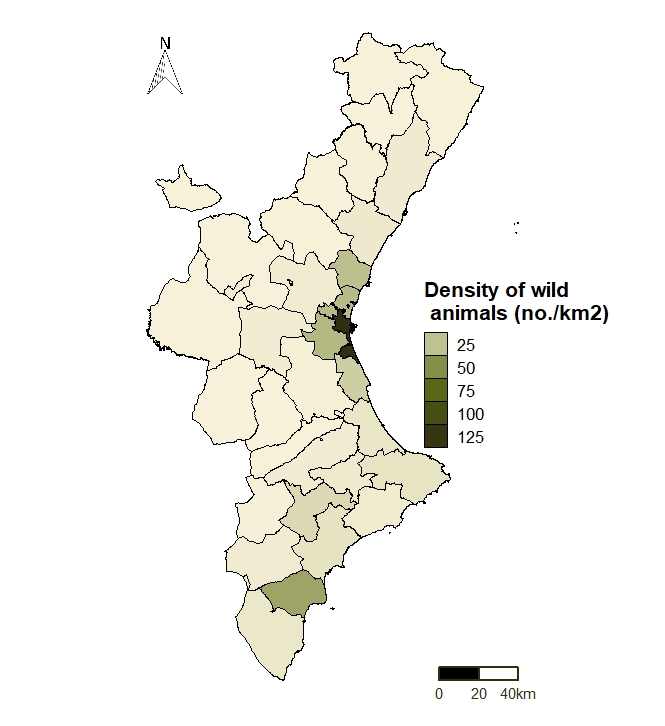

Supplement: Supplementary file 1 [file Data_Sheet_1.ZIP › i8.jpeg]

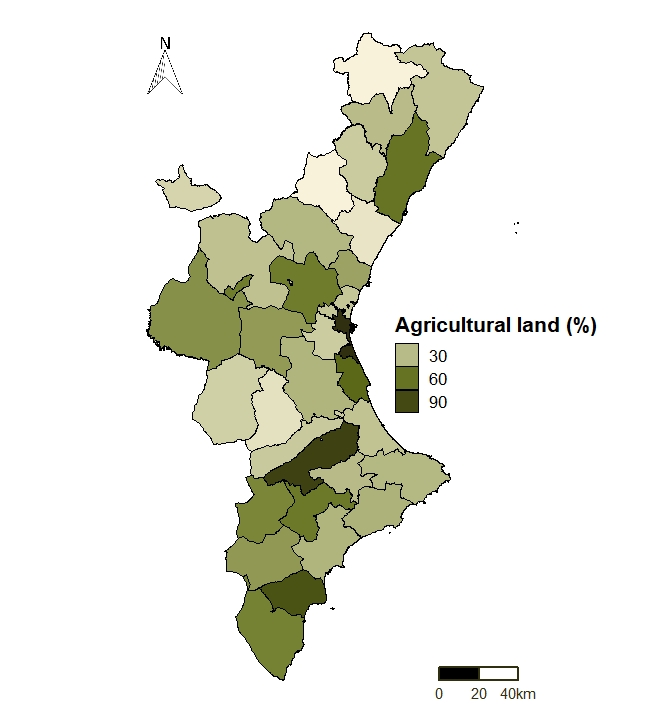

Supplement: Supplementary file 1 [file Data_Sheet_1.ZIP › i9.jpeg]
